# Supplementary material for: Linking genome size variation to population phenotypic variation within the rotifer, Brachionus asplanchnoidis
Source: Commun Biol. 2021 May 19;4:596. doi: 10.1038/s42003-021-02131-z (PMC8134563; doi:10.1038/s42003-021-02131-z)
Supplement: Supplementary file 2 — Supplementary Information [file 42003_2021_2131_MOESM2_ESM.pdf]

Supplementary Information for:

Linking genome size variation to population phenotypic variation within the rotifer, *Brachionus asplanchnoidis*

Claus-Peter Stelzer, Maria Pichler & Anita Hatheuer

Correspondence to: claus-peter.stelzer@uibk.ac.at

This file includes:

- Supplementary Methods
- Supplementary Figures 1 – 5
- Supplementary Tables 1 – 3
- Supplementary References

Supplementary Methods

Body (BS) and egg size (ES) measurements

Measurements of body size and egg size were done semi-automatically with a custom-made image analysis system. This system consisted of a simple, low-magnification inverted microscope (4x lens with a 160mm mechanical tube length) with an attached industrial monochrome digital camera with a resolution of 6 megapixels (PixeLINK®, model PL-B781F). The microscope was mounted under a motorized scanning table (Zaber, model ASR100B120B) with illumination from the top (Thorlabs LED lamp). The scanning table was equipped with a multiwell-microplate Insert, allowing to mount a 96-well plate. The scanning table was controlled by a custom program written in Labview 8.6 and NI Vision (National Instruments), which allowed controlling the movement of the scanning table at a precision of a few micrometers. Thus, samples were automatically evaluated by taking pictures at 20 pre-specified locations for each well, which covered the entire bottom of a well in a grid of four-by-five image sections (Fig. S1). The whole well was automatically reconstructed by digitally stitching the sections together in the correct orientation. This resulted in one large, high-resolution picture from each well (120 mega pixels), which was then automatically processed using NI Vision, also running within the Labview programming environment. Briefly, after the thresholding algorithm of NI Vision generated a binary image (i.e., an image consisting of black and white pixels only), it identified "particles" (i.e., contiguous nonzero pixels in an image) meeting certain size criteria (Fig. S2). A cropped picture of each particle (consisting of 10-500 thousand pixels) was saved for later analysis. At the same time, various metrics for particle classification were recorded and saved in csv-format. For size classification, we used several build-in functions of NI Vision to estimate body length, width, or area (maximum feret diameter, minimum feret diameter, area in pixels). For shape classification of particles, we considered four commonly used particle factor measurements implemented in the NI Vision software package (Elongation Factor, Compactness Factor, Heywood Circularity Factor, Type Factor) and four particle moment measurements (Moment of Inertia xx, xy, yy, and Hu Moment 1). More information on definition and formula used for calculation, see the National Instruments website (http://zone.ni.com/reference/en-XX/help/372916T-01/nivisionconcepts/particle_measurements/).

At this initial stage, the recorded particles did not only include well-separated females and eggs, but also objects that were not usable for analysis, such as detritus, aggregates of multiple eggs/females, or juvenile females (which sometimes hatched in the short period between culture harvest and fixation). Thus, we manually assigned the categories to each particle, e.g. egg, adult female, detritus particle. Manual assignment was facilitated by a custom Labview®-program, which stepped through the cropped pictures of all particles in a well and added a column to the csv-file indicating the particle

category. For later analyses, we only used eggs and females that were completely isolated and at suitable orientation for accurate measurement.

The raw data consisted of a csv-table for each well, which contained all objects as rows (i.e., eggs, females) and their size and shape characteristics as columns (see table). We also saved a cropped picture of each object, its respective position in the well, as well as the high-resolution picture of the whole well. The latter were not used in the subsequent analysis but were kept as backup. The csv tables were combined using a Matlab® program and a "master-table" containing information of all clone names and the links to their respective csv-files. Eventually, this resulted in one big table for each particle class (e.g., egg or female) with additionally generated columns for clone name, measurement date, and well identifier. After the raw data had been curated as described above, we calculated means of each metric for each rotifer clone.

Embryonic development time (EDT)

The duration of embryonic development was measured via time-lapse recording using a modified version of the above image analysis system, which allowed to simultaneously record hatching of ~100 embryos over the course of approximately one day. In the modified system, we used a lower magnification (1x magnification lens), which resulted in a larger field-of-view, covering almost the entire area of one well. Pictures of every egg were taken automatically in 30 min intervals by moving the motorized table from well to well. The time of hatching could then be reconstructed from the time stamps of two consecutive pictures, one before and one just after a hatching event. For more details on the setup of this system, see [1].

Measurements of the embryonic development time were done at 23°C, with clonal cultures acclimated to this temperature for at least one week growing at *ad libitum* food concentrations. Single freshly extruded asexual eggs (maximum age of 30 min since egg extrusion) were stripped from females and placed individually into the center of wells of a 96-well plate. Individual wells were topped-off with fresh medium and sealed with microscopic cover slips, to prevent evaporation during time-lapse monitoring. All hardware, including the laptop controlling the time-lapse recordings, was placed into an incubator at 23°C. On the following day, hatching success was checked by microscopic examination of the whole well plate. Hatching times were reconstructed by manual inspection of the digital pictures and their respective time stamps.

Our whole dataset on embryonic development times consisted of recordings from 2219 eggs. Some observations had to be filtered out as they were not unusable for the path analysis: accidentally picked male eggs (n = 18), unhatched eggs that did not hatch within >30 hours (n = 51), and eggs that were apparently lost during the transfer to the 96-well plate (n = 24). However, we did separately analyze

(1) whether male eggs differ in embryonic development time from female eggs, and (2) whether unhatched eggs differed in size from eggs of the remaining data set. For male eggs, we calculated the relative embryonic development time as the ratio of EDT_male to mean EDT of females of the same clone. For unhatched eggs, we calculated the relative egg size (volume), as the ratio of EV_unhatched to mean egg size of hatched eggs of the same clone. For each egg, we recorded the dependent variable 'embryonic development time' and the independent variables 'egg size' (area in pixels, minimum feret diameter, maximum feret diameter. For more information, see the National Instruments website (http://zone.ni.com/reference/en-XX/help/372916T-01/nivisionconcepts/particle_measurements/).

Supplementary Figure 1: Image acquisition.

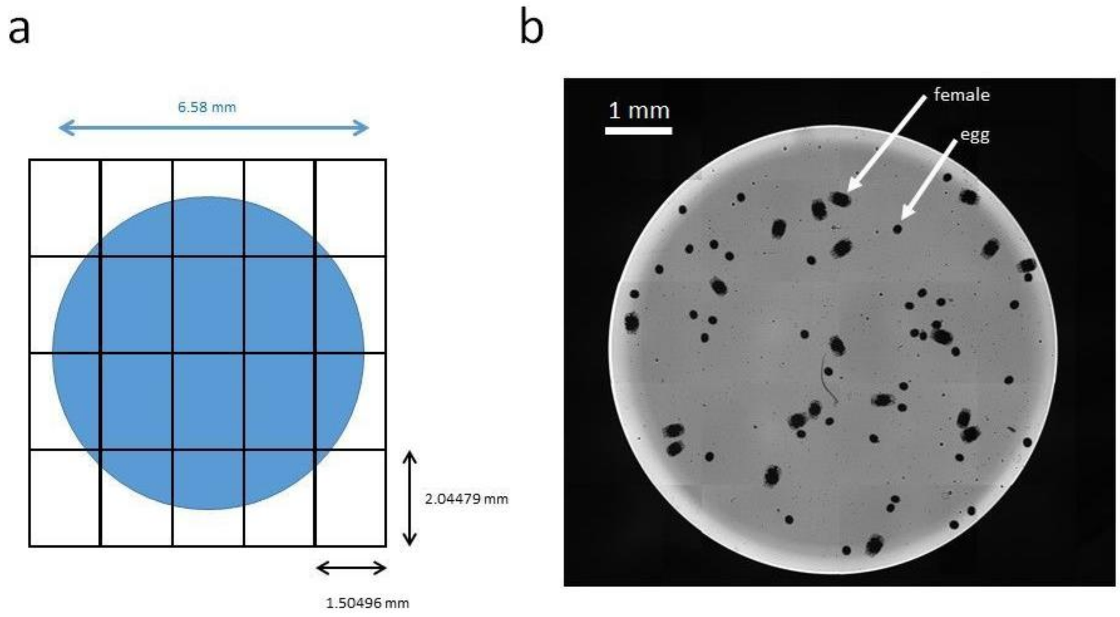

a Grid of 20 individual pictures covering the bottom of well of a 96-well plate. **b** Example of a compound picture of a well, obtained by stitching 20 pictures together.

Supplementary Figure 2: Image analysis workflow.

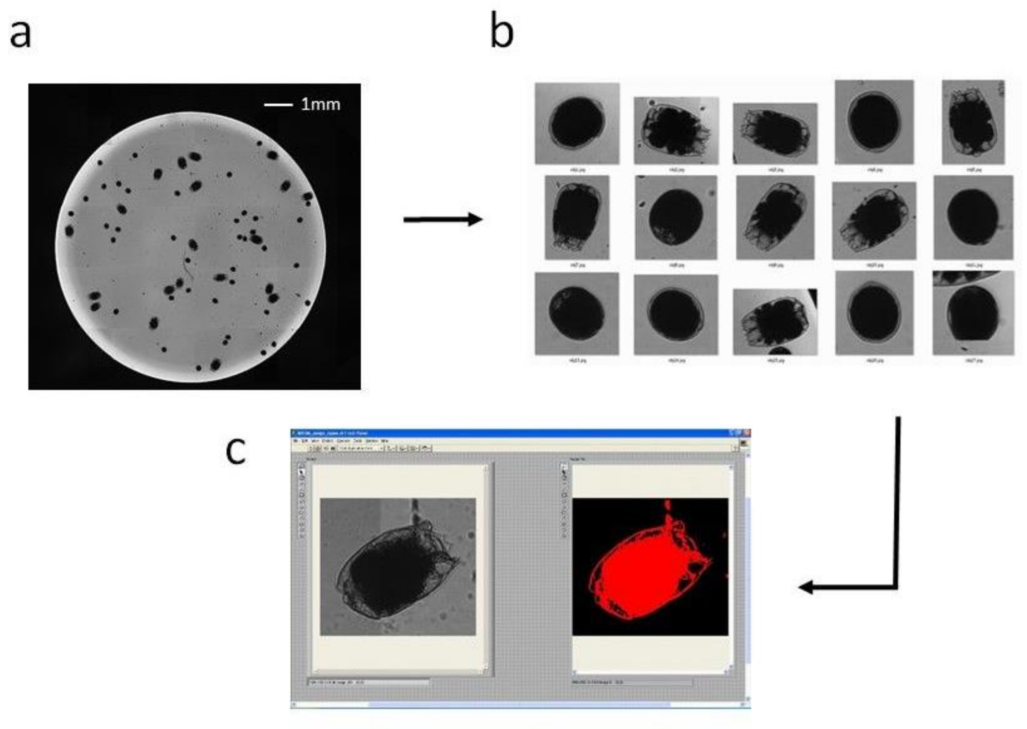

a Picture of an entire well of a 96-well plate. **b** cropped pictures of “particles” sampled from the well. **c** Example of thresholding of a particle (here: a female rotifer) by NI Vision. After thresholding, various size and shape parameters are extracted and stored in csv-files (not shown).

Supplementary Figure 3: Body size dimorphism in the OHJ-population of *B. asplanchnoidis*.

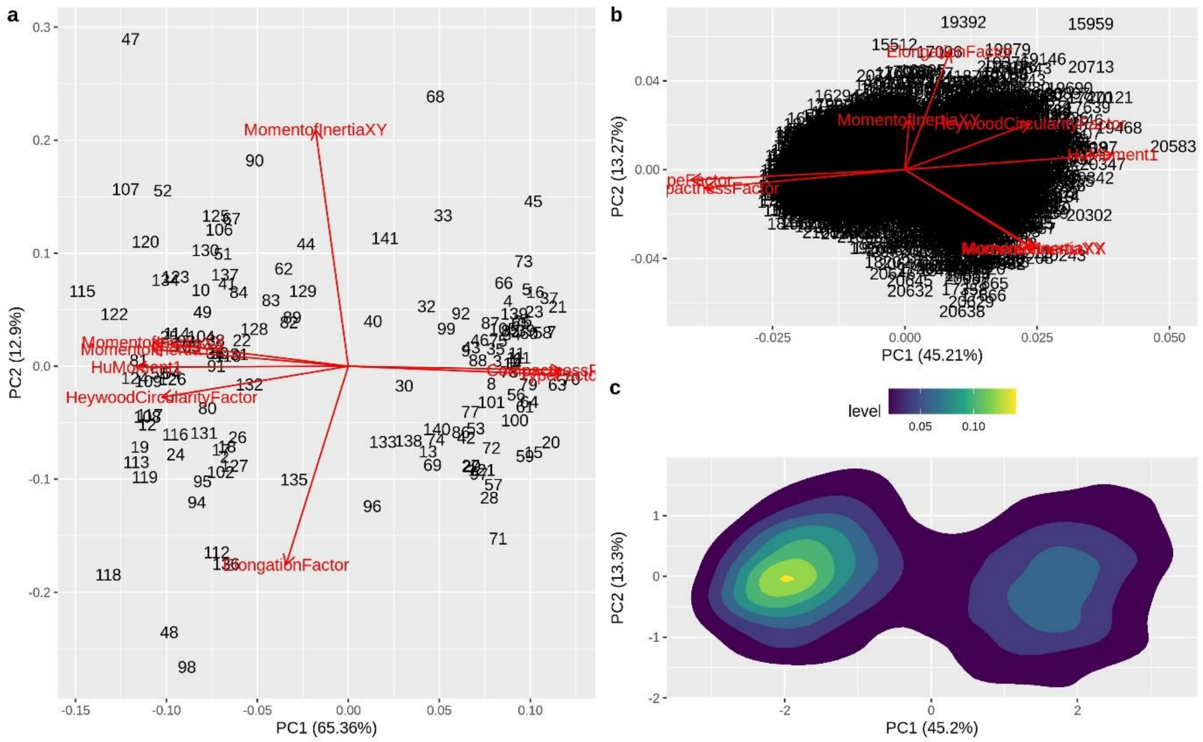

a Principal components analysis on clone means of shape parameters. **b** and **c** PCA based on data from individual females (n = 5644). Please note that the classification based on individual females (**b**, **c**) is just shown here for demonstration, but that all correlation analyses in the main text are based on clone means (**a**).

Supplementary Figure 4: Relationship between egg size and body size.

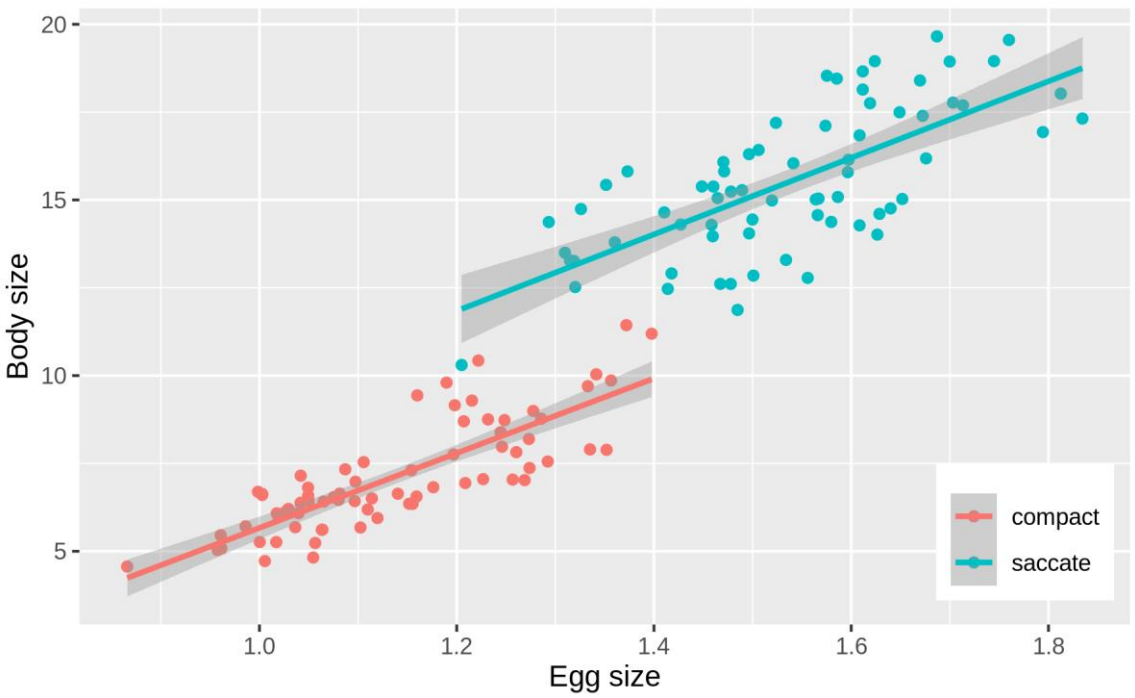

Volumes are given in 10^6 femtoliters for both morphotypes (Compact morph: n = 72, saccate morph: n = 69).

Supplementary Figure 5: Male eggs and unhatched female eggs compared to female eggs of the remaining dataset.

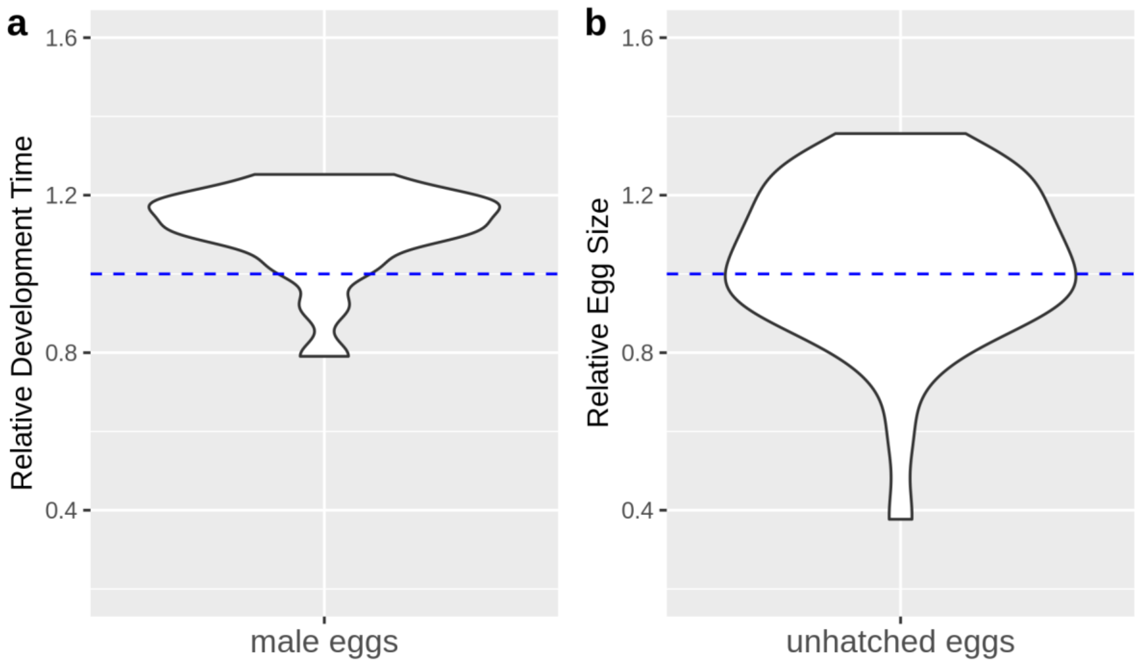

a Development time of male eggs relative to the mean EDT of females of the same clone. **b** Egg volume of unhatched eggs relative to egg volume of unhatched female eggs of the same clone.

Supplementary Table 1: Structural equation model of the direct and indirect (via egg size) effects of genome size on embryonic development time.

eggsize ~ genomesize

	Estimate	z-value	P(>|z|)
genomesize	0.262	12.176	0.000

R^2 = 0.069

devtime ~ eggsize + genomesize

	Estimate	z-value	P(>|z|)
eggsize	0.214	9.721	0.000
genomesize	0.163	7.376	0.000

R^2 = 0.091

	Estimate	z-value	P(>|z|)
indirect	0.056	7.597	0.000
direct	0.163	7.376	0.000
total	0.219	10.049	0.000

Supplementary Table 2: Influence of morphotype and genome size on sexual propensity. GLM model and analysis of deviance. This table corresponds to the data displayed in Fig. 3a,b.

	Estimate	Std. Error	z value	Pr(>|z|)	
Intercept	3.723	0.883	4.216	2.49E-05	***
morphotype	1.898	0.202	9.409	< 2e-16	***
genome size	-0.001	0.002	-0.896	0.37	

	df	Deviance	df (Deviance)	Pr(>Chi)	
NULL		239.65	134		
morphotype	1	163.73	133	<2e-16	***
genomesize	1	163.08	132	0.419	

Supplementary Table 3: Influence of morphotype and genome size on population growth. GLM model and analysis of deviance. This table corresponds to the data displayed in Fig. 3 c,d.

	**Estimate**	**Std. Error**	**t value**	**Pr(>|t|)**	
Intercept	0.3406812	0.241463	1.411	0.161	
morphotype	-1.016312	0.2876461	-3.533	5.68E-04	***
genomesize	-0.00208	0.0004969	-4.186	5.17E-05	***
morphotype x genomesize	0.0021419	0.0005873	3.647	3.82E-04	***

	**df**	**Deviance**	**df (Deviance)**	**Pr(>Chi)**	
NULL					
morphotype	1	0.003795	133	0.448	
genomesize	1	0.028626	132	0.037	*
morphotype x genomesize	1	0.08791	131	2.60E-04	***

Supplementary References

1. Stelzer, C.P. (2017). Extremely short diapause in rotifers and its fitness consequences. Hydrobiologia *796*, 255-264.
