# Supplementary material for: Linking genome size variation to population phenotypic variation within the rotifer, Brachionus asplanchnoidis
Source: Commun Biol. 2021 May 19;4:596. doi: 10.1038/s42003-021-02131-z (PMC8134563; doi:10.1038/s42003-021-02131-z)
Supplement: Supplementary file 3 — Descriptions of Additional Supplementary Files [file 42003_2021_2131_MOESM3_ESM.pdf]

Descriptions of Additional Supplementary Files

Supplementary Data 1
Description: Raw data of automated body and egg size measurements.

Supplementary Data 2
Description: Morphotype classification of different rotifer clones based on Principle Component Analysis of body shape parameters.

Supplementary Data 3
Description: Raw data on embryonic development time.

Supplementary Data 4
Description: Data on population growth and male ratio.
