# Supplementary material for: Linking genome size variation to population phenotypic variation within the rotifer, Brachionus asplanchnoidis
Source: Commun Biol. 2021 May 19;4:596. doi: 10.1038/s42003-021-02131-z (PMC8134563; doi:10.1038/s42003-021-02131-z)
Supplement: Supplementary file 8 — Reporting Summary [file 42003_2021_2131_MOESM8_ESM.pdf]

nature research

Corresponding author(s): Claus-Peter Stelzer

Last updated by author(s): Mar 18, 2021

Reporting Summary

Nature Research wishes to improve the reproducibility of the work that we publish. This form provides structure for consistency and transparency in reporting. For further information on Nature Research policies, see our Editorial Policies and the Editorial Policy Checklist.

Statistics

For all statistical analyses, confirm that the following items are present in the figure legend, table legend, main text, or Methods section.

n/a	Confirmed	
☐	☒	The exact sample size (*n*) for each experimental group/condition, given as a discrete number and unit of measurement
☐	☒	A statement on whether measurements were taken from distinct samples or whether the same sample was measured repeatedly
☐	☒	The statistical test(s) used AND whether they are one- or two-sided<br>*Only common tests should be described solely by name; describe more complex techniques in the Methods section.*
☐	☒	A description of all covariates tested
☐	☒	A description of any assumptions or corrections, such as tests of normality and adjustment for multiple comparisons
☐	☒	A full description of the statistical parameters including central tendency (e.g. means) or other basic estimates (e.g. regression coefficient) AND variation (e.g. standard deviation) or associated estimates of uncertainty (e.g. confidence intervals)
☐	☒	For null hypothesis testing, the test statistic (e.g. *F*, *t*, *r*) with confidence intervals, effect sizes, degrees of freedom and *P* value noted<br>*Give P values as exact values whenever suitable.*
☐	☐	For Bayesian analysis, information on the choice of priors and Markov chain Monte Carlo settings
☐	☐	For hierarchical and complex designs, identification of the appropriate level for tests and full reporting of outcomes
☐	☒	Estimates of effect sizes (e.g. Cohen's *d*, Pearson's *r*), indicating how they were calculated

Our web collection on statistics for biologists contains articles on many of the points above.

Software and code

Policy information about availability of computer code

Data collection	National Instruments Labview 8.6 and Vision Development module<br>Matlab 9.4 (2018a)
Data analysis	R (version 4.0.2) and packages, as reported in Materials and Methods

For manuscripts utilizing custom algorithms or software that are central to the research but not yet described in published literature, software must be made available to editors and reviewers. We strongly encourage code deposition in a community repository (e.g. GitHub). See the Nature Research guidelines for submitting code & software for further information.

Data

Policy information about availability of data

All manuscripts must include a data availability statement. This statement should provide the following information, where applicable:

- Accession codes, unique identifiers, or web links for publicly available datasets
- A list of figures that have associated raw data
- A description of any restrictions on data availability

All data supporting the findings of this study are available within the paper and its supplementary information files

Field-specific reporting

Ecological, evolutionary & environmental sciences study design

All studies must disclose on these points even when the disclosure is negative.

Study description	Measurements of body size and egg size in Lugol-fixed rotifers. Measurements on egg hatching times, population growth, and male frequency in live rotifers cultured in the laboratory.
Research sample	Clonally cultured rotifers which were derived (hatched) from a natural population, and their F1 (outbred) sexual offspring
Sampling strategy	Sample sizes were chosen for each measurement type based on previous experiments/experience with this model organism
Data collection	Data collection for each measured variable is described in detail in Materials and Methods and in the Supplementary Information
Timing and spatial scale	Data collection took place between 2015 and 2018, within 1-2 months after a clone had been established in the lab. All measurements were done in the laboratory.
Data exclusions	In this study we only used clones from a single panmictic population (Obere Halbjochlacke). Clones from the same species (B. asplanchnoidis) but different populations were excluded.
Reproducibility	Different measurements were done continuously using highly standardized protocols. Measurements on the basic unit (rotifer clone) were spread out over time.
Randomization	There were no groups/treatments in this dataset. We basically included those clones that we could establish from this population.
Blinding	Blinding was used during data collection as the person obtaining the measurement did not know the independent variable (genome size). In the data analysis we used automated algorithms, e.g. for calculation of clonal means, across the entire dataset.

Did the study involve field work? ☐ Yes ☒ No

Reporting for specific materials, systems and methods

We require information from authors about some types of materials, experimental systems and methods used in many studies. Here, indicate whether each material, system or method listed is relevant to your study. If you are not sure if a list item applies to your research, read the appropriate section before selecting a response.

Materials & experimental systems

n/a	Involved in the study
☒	☐ Antibodies
☒	☐ Eukaryotic cell lines
☒	☐ Palaeontology and archaeology
☐	☒ Animals and other organisms
☒	☐ Human research participants
☒	☐ Clinical data
☒	☐ Dual use research of concern

Methods

n/a	Involved in the study
☒	☐ ChIP-seq
☒	☐ Flow cytometry
☒	☐ MRI-based neuroimaging

Animals and other organisms

Policy information about studies involving animals; ARRIVE guidelines recommended for reporting animal research

Laboratory animals	Brachionus asplanchnoidis, females, parthenogenetic reproduction (apomixis) with occasional male production
Wild animals	Animals were born in the lab
Field-collected samples	Animals were born in the lab
Ethics oversight	No ethical approval required for this animal group

Note that full information on the approval of the study protocol must also be provided in the manuscript.
